# Adult‐onset CD8‐predominant hemispheric inflammatory epilepsy: Expanding the spectrum of post‐infectious and Rasmussen‐like encephalitis

**DOI:** 10.1002/epd2.70281

**Published:** 2026-07-04

**Authors:** Ramya Raghupathi

**Affiliations:** ^1^ Hospital of University of Pennsylvania Philadelphia Pennsylvania USA

Adult‐onset hemispheric inflammatory epilepsy is rare and diagnostically challenging. Classic Rasmussen encephalitis (RE) is predominantly a pediatric disorder characterized by progressive hemispheric atrophy and cytotoxic T‐cell–mediated inflammation. We describe two immunocompetent adults presenting with drug‐resistant focal epilepsy and progressive unilateral cortical abnormalities. Both highlight a possible adult‐onset spectrum of cytotoxic hemispheric inflammatory epilepsy that overlaps with, but does not fully satisfy, classical RE criteria, with implications for biopsy timing and immunotherapy selection.

Rasmussen encephalitis (RE) has an estimated incidence of 2.4 cases per 10^7^ people aged 18 years or younger per year.^1^ Adult‐onset RE is rare and often atypical, frequently lacking epilepsia partialis continua, early hemiparesis, or complete Bien diagnostic criteria.^2^ The majority of late‐onset cases fulfill Part B rather than Part A Bien criteria, reflecting the diagnostic delay inherent to the attenuated adult phenotype; immunomodulatory therapy achieves a clinically meaningful response in approximately 61% of late‐onset patients even when initiated late (Data S1).^3^ Increasing recognition of post‐infectious autoimmune epilepsy, particularly following herpes simplex virus (HSV) encephalitis, where anti‐NMDAR encephalitis may emerge weeks after viral clearance,^4^ raises the possibility that cytotoxic inflammatory hemispheric epilepsy in adults may represent a broader immunopathologic spectrum than classical RE alone.

## 1 CASE 1

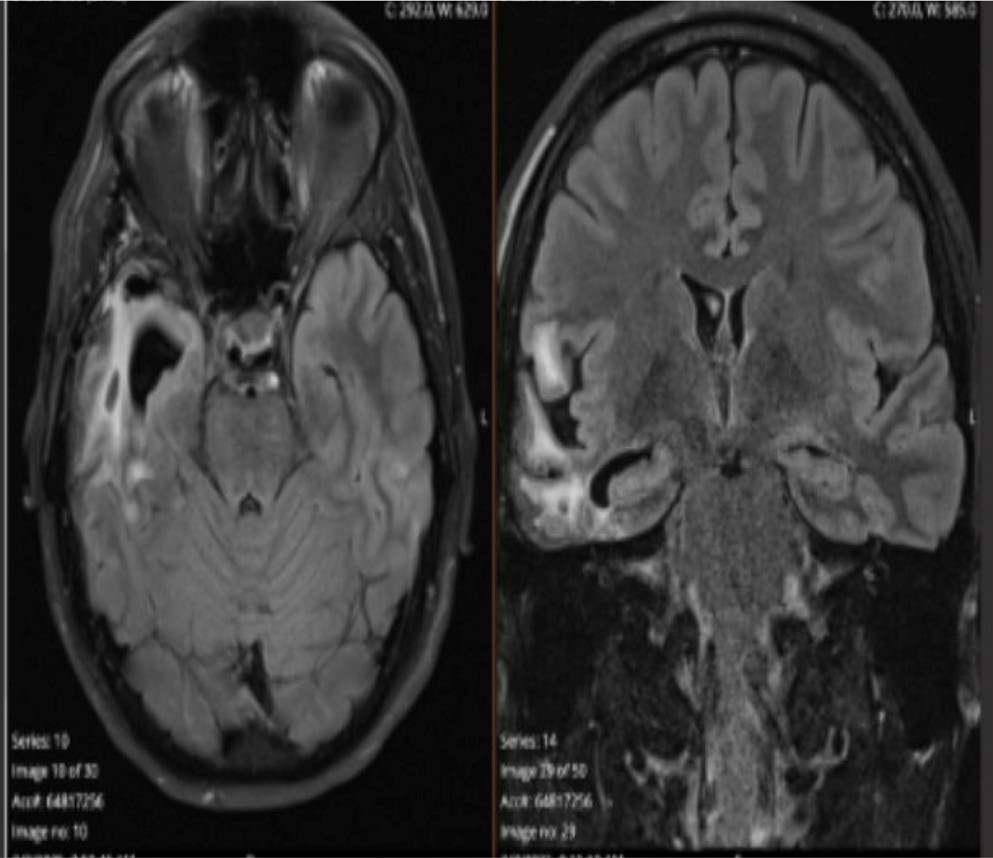

A 33‐year‐old immunocompetent male presented in 2022 with convulsive status epilepticus. MRI demonstrated a right temporal lesion; spectroscopy prompted tumor board concern, leading to craniotomy. Pathology revealed necrotizing encephalitis with perivascular lymphocytic inflammation, reactive gliosis, and focal necrosis. HSV immunohistochemistry was equivocal; empiric valacyclovir was prescribed. He remained seizure‐free until mid‐2023, when focal aware seizures emerged. Serial MRIs (2024–2025) showed progressive FLAIR abnormalities and curvilinear enhancement extending into the right perisylvian cortex, inconsistent with active HSV or neoplasm. Four serial CSF examinations were noninflammatory with negative viral PCR. Comprehensive autoimmune panels (anti‐NMDAR, LGI1, CASPR2, AMPAR, GABA‐A/B, DPPX, GAD65, MOG, and others) were entirely negative in serum and CSF. Multidisciplinary consensus favored post‐infectious or secondary autoimmune inflammatory injury.

## 2 CASE 2

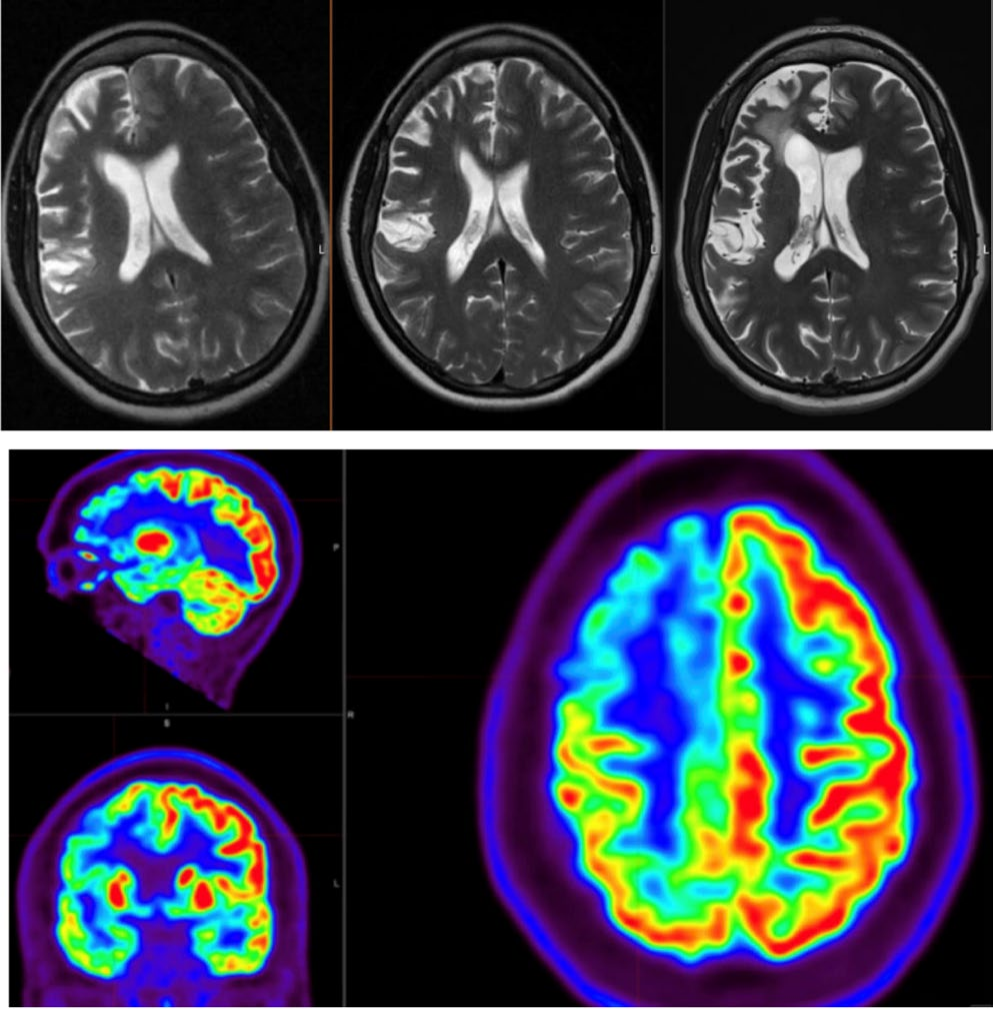

A 31‐year‐old woman developed drug‐resistant focal epilepsy at age 29, with focal bilateral tonic–clonic, focal impaired consciousness, and gelastic seizures. Serial MRI over 15 months demonstrated progressive right hemispheric volume loss involving the frontal lobe, insula, perisylvian cortex, basal ganglia, and cerebral peduncle. FDG‐PET showed extensive right frontal, mesial temporal, and thalamic hypometabolism. CSF revealed oligoclonal bands but was otherwise noninflammatory; serum autoimmune panels were negative. Stereotactic biopsy demonstrated CD8‐predominant T‐cell infiltration in gray and white matter without viral etiology, demyelination, or antibody‐mediated pathology, meeting probable Bien criteria for late‐onset RE. Following treatment of latent tuberculosis, rituximab was initiated; the patient was clinically stable at last follow‐up (January 2026).

The pathologic hallmark of RE is CD8^+^ cytotoxic T‐cell–mediated injury, independent of antibody‐mediated mechanisms, explaining the seronegative profile that renders even comprehensive autoimmune panels uninformative.^3^ Case 2 exemplifies this directly.

Post‐infectious autoimmune encephalitis following HSV is best characterized by anti‐NMDAR encephalitis emerging weeks after viral clearance, reported in up to 27% of HSV encephalitis survivors in prospective series.^2^ In Case 1, equivocal HSV immunohistochemistry and a history of recurrent herpes labialis raise the possibility that a subclinical viral exposure initiated chronic T‐cell–predominant inflammatory injury, a phenotype distinct from anti‐NMDAR encephalitis, where the effector is cytotoxic rather than humoral and serologic testing is uninformative. Late‐onset RE is clinically heterogeneous: among 102 published cases, epilepsia partialis continua occurred in only 31% and neurologic deficits appeared on average 3 years after onset.^3^ Gambardella et al.^5^ described a further variant, limited chronic focal encephalitis, in which the inflammatory process remained circumscribed over more than two decades without progressing to diffuse hemispheric atrophy, confirming that restricted distribution should not preclude the diagnosis. Case 2 fits the late‐onset profile. The Bien criteria are met at the probable level (Table 1).

**TABLE 1:** Comparative clinical, imaging, and immunopathologic features.

Feature	Case 1—Post‐HSV inflammatory encephalitis	Case 2—Probable late‐onset RE
Age/sex	33/M	31/F
Seizure onset	Age 33; convulsive status epilepticus	Age 29; focal aware, FIC, FBTC, gelastic
Seizure type at presentation	Status epilepticus; no prodrome or focal deficit	Nocturnal FBTC → daytime FIC and gelastic seizures
Imaging pattern	Progressive right temporal → frontal operculum/perisylvian FLAIR signal, curvilinear enhancement, microhemorrhage, BBB disruption	Progressive right hemispheric atrophy: frontal, insula, perisylvian, postcentral, BG, IC, peduncle
Lesion lateralization	Right hemisphere (progressive multifocal)	Right hemisphere (unilateral diffuse)
FDG‐PET	Not performed	Extensive right frontal + mesial temporal + thalamic hypometabolism
EEG	Right temporal slowing; right frontotemporal IEDs (sleep‐predominant); clinical events without EEG correlate	Right frontocentral/hemispheric IEDs, LPDs, LRDA; right hemispheric ictal onset
CSF	Repeatedly noninflammatory; normal cell count, protein, OB; negative HSV/VZV/JC PCR	Oligoclonal bands (2 CSF / 0 serum); otherwise noninflammatory
Serum autoantibodies	Anti‐NMDAR negative; ENS2 panel pending	MOG, GAD65, PCCA/ANNA, GABA‐A, ENS2/ENC2 — all negative
Index surgical/biopsy pathology	Necrotizing encephalitis: perivascular + intraparenchymal lymphocytic infiltration, focal necrosis, gliosis, microgliosis; equivocal HSV IHC (2/3 stains negative); no intranuclear inclusions	CD8‐predominant T‐cell infiltrate, gray + white matter; reactive gliosis and microgliosis; no viral etiology, demyelination, PML, or MOGAD
CD8 predominance confirmed	Not formally established (repeat biopsy pending)	Yes
Confirmed viral infection	No (equivocal IHC; negative serial CSF PCR)	No
Presumed mechanism	Post‐infectious / secondary cytotoxic autoimmune inflammatory injury	CD8+ cytotoxic T‐cell–mediated cortical injury
Diagnostic classification	Chronic inflammatory encephalitis, etiology uncertain; RE spectrum possible	Probable late‐onset Rasmussen's encephalitis (Bien criteria; atypical)
ASM response	Drug‐resistant; focal aware seizures persisting on therapy	Drug‐resistant (lamotrigine, levetiracetam, clobazam)
Immunotherapy	Empiric immunotherapy planned pending repeat biopsy	IV methylprednisolone + IVIG (peri‐biopsy; unclear benefit); rituximab initiated (2 doses; well tolerated)
Antiviral treatment	Empiric valacyclovir (post‐surgical; ongoing ID follow‐up)	Not applicable
Surgical intervention	Right temporal craniotomy (December 2022); subsequent disease progression	None (lesion extent; absent hemiparesis); neuromodulation under consideration
Follow‐up status	Progressive imaging; stable neurologic exam; repeat biopsy planned	Clinically stable on rituximab (January 2026); seizure burden monitoring ongoing

Abbreviations: ASM, antiseizure medication; BBB, blood–brain barrier; BG, basal ganglia; FBTC, focal bilateral tonic–clonic; FIC, focal impaired consciousness; HSV, herpes simplex virus; IC, internal capsule; ID, infectious disease; IED, interictal epileptiform discharge; IHC, immunohistochemistry; LPD, lateralized periodic discharge; LRDA, lateralized rhythmic delta activity; OB, oligoclonal bands; PML, progressive multifocal leukoencephalopathy; RE, Rasmussen's encephalitis.

Together, these cases suggest adult hemispheric inflammatory epilepsy exists on a continuum from established RE to post‐infectious cytotoxic encephalitis, connected by shared immunopathology. Both underscore the diagnostic primacy of tissue in seronegative inflammatory epilepsy. Earlier biopsy may enable earlier treatment decisions before cumulative cortical injury accrues, though this remains hypothesis‐generating. The hypothesis, consistent with published RE literature but not directly demonstrated here, that early repeat biopsy in adults with progressive hemispheric inflammatory changes, negative CSF, and seronegative evaluation may enable earlier treatment decisions, since imaging progression may outpace neurologic decline.^6^

Regarding immunotherapy, tacrolimus and IVIG remain best‐studied in RE, with prospective data demonstrating delayed deterioration but not reversal of pharmacoresistance.^1^ Rituximab, initiated in Case 2, represents an emerging option; six‐month follow‐up is insufficient to assess efficacy.^7^, ^8^

Limitations include the two‐case design, unconfirmed CD8 predominance in Case 1, and insufficient rituximab follow‐up precluding causal conclusions. Adult‐onset progressive hemispheric inflammatory epilepsy with CD8‐predominant infiltration may represent a post‐infectious or autoimmune spectrum overlapping with RE, needing prospective validation. These cases underscore the importance of longitudinal imaging, early tissue diagnosis, and multidisciplinary evaluation in adults with progressive focal epilepsy and unilateral cortical abnormalities.

## AUTHOR CONTRIBUTIONS

**Ramya Raghupathi:** Conceptualization; investigation; data curation; formal analysis; writing – original draft; writing – review and editing; visualization; supervision; project administration.

## FUNDING INFORMATION

This work received no specific grant from any funding agency in the public, commercial, or not‐for‐profit sectors.

## CONFLICT OF INTEREST STATEMENT

The author declares that there is no conflict of interest.

## INFORMED CONSENT

Verbal consent was obtained for the chart review.

**Test yourself**

1. Which of the following best explains why comprehensive autoimmune antibody panels are typically negative in Rasmussen encephalitis?
  - The immune response is IgA‐mediated rather than IgG
  - The pathologic mechanism is CD8^+^ cytotoxic T‐cell–mediated injury, independent of antibody‐mediated pathways
  - Patients with RE have generalized immunosuppression
  - Current panels do not test for NMDAR or LGI1 antibodies
2. Regarding immunotherapy in late‐onset Rasmussen encephalitis, which of the following statements is most accurate based on available evidence?
  - Hemispherectomy is the only proven treatment in adults
  - Corticosteroids alone are sufficient for long‐term seizure control
  - Tacrolimus and IVIG have prospective data showing delayed deterioration, though not reversal of pharmacoresistance
  - Rituximab has robust long‐term trial data supporting its use as first‐line therapy
3. Which of the following features is most useful in distinguishing adult‐onset cytotoxic hemispheric inflammatory epilepsy from anti‐NMDAR encephalitis following HSV infection?
  - Presence of oligoclonal bands in CSF
  - Positive serum anti‐NMDAR antibodies and humoral effector mechanism versus seronegative cytotoxic T‐cell predominance on biopsy
  - Bilateral hemispheric involvement on MRI
  - Response to antiepileptic monotherapy

*Answers may be found in the* *Supporting Information*

## Supporting information


Data S1

Data S2

Appendix S1.

## Data Availability

The data that support the findings of this study are available from the corresponding author upon reasonable request.
