# Supplementary material for: Adult‐onset CD8‐predominant hemispheric inflammatory epilepsy: Expanding the spectrum of post‐infectious and Rasmussen‐like encephalitis
Source: Epileptic Disord. 2026 Jul 4;28(5):1679–82. doi: 10.1002/epd2.70281 (PMC13695466; doi:10.1002/epd2.70281)
Supplement: Supplementary file 1 [file EPD2-28-1679-s003.pdf]

Adult-Onset CD8-Predominant Hemispheric Inflammatory Epilepsy

Expanding the Spectrum of Post-Infectious and Rasmussen-like Encephalitis

Epileptic Disorders · 2026 | **Raghupathi R**

LEARNING OBJECTIVES

1. Recognize adult-onset hemispheric inflammatory epilepsy spectrum
2. Understand why seronegative panels don't exclude inflammatory epilepsy
3. Apply Bien diagnostic criteria in adults
4. Select appropriate immunotherapy strategies

BACKGROUND

Classic Rasmussen Encephalitis (RE) is predominantly a pediatric disorder characterized by progressive hemispheric atrophy and cytotoxic T-cell–mediated inflammation. Adult-onset RE is rare, often atypical, and diagnostically challenging.

2.4

cases per 10^7 people ≤18 yr/year

Estimated RE incidence

~61%

of late-onset patients respond to immunotherapy

Even when initiated late

31%

of adult RE have epilepsia partialis continua

Much lower than in children

Adult RE Phenotype

Frequently lacks epilepsia partialis continua, early hemiparesis, or full Bien Part A criteria

Post-infectious Risk

Anti-NMDAR encephalitis emerges in up to 27% of HSV encephalitis survivors weeks after viral clearance

Gambardella Variant

Limited chronic focal encephalitis can remain circumscribed >2 decades — restricted distribution ≠ exclusion

BIEN DIAGNOSTIC CRITERIA · Applying to Adult Cases

PART A · Typical / Pediatric

- Seizure onset before age 10
- Unihemispheric focal EEG slowing + epileptiform activity
- Progressive unihemispheric atrophy on MRI
- Histopathology: T-cell dominated encephalitis
- Typically fulfills full criteria

PART B · Probable / Adult

- Focal seizures ± epilepsia partialis continua
- Unilateral EEG slowing + epileptiform activity
- Progressive unilateral atrophy on MRI (less strict)
- Histopathology: T-cell encephalitis + activated microglia ± astrogliosis
- *Most adult cases fulfill Part B, not Part A*

THE DIAGNOSTIC ROLE OF BIOPSY

WHEN TO BIOPSY?

- Progressive unilateral cortical changes on serial MRI
- Negative comprehensive autoimmune panel in serum and CSF
- Drug-resistant focal epilepsy without alternative diagnosis
- Negative CSF inflammatory markers and viral PCR
- Clinical or imaging progression despite initial therapy

WHAT BIOPSY PROVIDES

- Confirms CD8-predominant T-cell infiltration
- Excludes mimics: lymphoma, active viral encephalitis, demyelination, MOGAD, PML
- Guides immunotherapy selection
- Enables earlier treatment before cumulative cortical injury accrues
- Supports classification for longitudinal management

"Imaging progression may outpace neurologic decline — earlier biopsy may enable earlier treatment decisions."

BIOPSY EXCLUDES THESE MIMICS:

CNS Lymphoma	Active HSV Encephalitis	MOGAD
Demyelination (MS)	PML	Antibody-mediated encephalitis

IMMUNOTHERAPY IN ADULT RE

Agent	Evidence Level	Effect	Limitation
Tacrolimus	Prospective RCT	Delays deterioration	Does NOT reverse pharmacoresistance
IVIG	Prospective data	Delays deterioration	Does NOT reverse pharmacoresistance
IV Methylprednisolone	Case series	Short-term benefit	Unclear long-term value
Rituximab	Emerging; case-level	Promising (Case 2 stable)	Insufficient long-term data

2 3 ✓

A PROPOSED SPECTRUM

Post-Infectious Cytotoxic	Overlap Zone	Classic RE (Adult/Pediatric)
Case 1		Case 2

SHARED FEATURES ACROSS THE SPECTRUM

- CD8+ cytotoxic T-cell immunopathology
- Seronegative autoimmune panels (expected)
- Drug-resistant focal epilepsy
- Progressive unilateral cortical injury
- Tissue biopsy required for diagnosis
- Adult attenuated phenotype (Bien Part B)

Adult hemispheric inflammatory epilepsy exists on a continuum requiring prospective validation.

KEY TAKE-HOME MESSAGES

1

Think Beyond Pediatrics

Adult hemispheric inflammatory epilepsy exists on a spectrum from post-infectious cytotoxic encephalitis to classic RE.

2

Seronegative ≠ Non-inflammatory

Negative autoimmune panels are expected in CD8-mediated disease. Do not stop the workup — proceed to biopsy.

3

Biopsy Early

In adults with progressive unilateral cortical changes and negative serology, tissue diagnosis should not be deferred.

4

Apply Bien Criteria Carefully

Most adult cases meet Part B (probable), not Part A. Attenuated phenotype is typical of late-onset RE.

5

Immunotherapy Can Help

~61% of late-onset RE patients respond meaningfully even with delayed initiation of immunomodulatory therapy.

6

Multidisciplinary Evaluation

Neurology, neuroradiology, neuropathology, infectious disease, and epilepsy surgery input all contribute to diagnosis.

Raghupathi R. Epileptic Disorders. 2026. DOI: 10.1002/epd2.70281
