# Supplementary material for: Adult‐onset CD8‐predominant hemispheric inflammatory epilepsy: Expanding the spectrum of post‐infectious and Rasmussen‐like encephalitis
Source: Epileptic Disord. 2026 Jul 4;28(5):1679–82. doi: 10.1002/epd2.70281 (PMC13695466; doi:10.1002/epd2.70281)
Supplement: Supplementary file 2 [file EPD2-28-1679-s001.docx]

Case 1

**Figure 1A.** Serial MRI findings in Case 1 (33-year-old male with post-infectious inflammatory encephalitis). Axial FLAIR sequences demonstrate progressive right temporal to frontal opercular and perisylvian signal abnormality with curvilinear enhancement, microhemorrhage, and blood-brain barrier disruption over the 2024–2025 surveillance period. Findings are inconsistent with active HSV encephalitis or neoplasm and are most consistent with ongoing cytotoxic inflammatory injury.

Case 2

**Figure 1B.** Serial MRI findings in Case 2 (31-year-old female with probable late-onset Rasmussen’s encephalitis). Axial FLAIR sequences over 15 months demonstrate progressive right hemispheric volume loss involving the frontal lobe, insula, perisylvian cortex, and postcentral gyrus, with extension to the basal ganglia, internal capsule, and cerebral peduncle. The pattern of unilateral hemispheric atrophy is consistent with probable Bien criteria for late-onset Rasmussen’s encephalitis.

**Figure 1C.** FDG-PET findings in Case 2. Axial and coronal FDG-PET images demonstrate extensive right frontal, mesial temporal, and thalamic hypometabolism. The pattern of predominantly right hemispheric hypometabolism correlates with the regions of structural atrophy on MRI and with the patient’s EEG-confirmed right hemispheric ictal onset.

**Supplementary Table 1. Comparative Clinical, Imaging, and Immunopathologic Features**

| **Feature** | **Case 1 — Post-HSV Inflammatory Encephalitis** | **Case 2 — Probable Late-Onset RE** |
| --- | --- | --- |
| **Age / sex** | 33M | 31F |
| **Seizure onset** | Age 33; convulsive status epilepticus | Age 29; focal aware, FIC, FBTC, gelastic |
| **Seizure type at presentation** | Status epilepticus; no prodrome or focal deficit | Nocturnal FBTC → daytime FIC and gelastic seizures |
| **Imaging pattern** | Progressive right temporal → frontal operculum/perisylvian FLAIR signal, curvilinear enhancement, microhemorrhage, BBB disruption | Progressive right hemispheric atrophy: frontal, insula, perisylvian, postcentral, BG, IC, peduncle |
| **Lesion lateralization** | Right hemisphere (progressive multifocal) | Right hemisphere (unilateral diffuse) |
| **FDG-PET** | Not performed | Extensive right frontal + mesial temporal + thalamic hypometabolism |
| **EEG** | Right temporal slowing; right frontotemporal IEDs (sleep-predominant); clinical events without EEG correlate | Right frontocentral/hemispheric IEDs, LPDs, LRDA; right hemispheric ictal onset |
| **CSF** | Repeatedly non-inflammatory; normal cell count, protein, OB; negative HSV/VZV/JC PCR | Oligoclonal bands (2 CSF / 0 serum); otherwise non-inflammatory |
| **Serum autoantibodies** | Anti-NMDAR, LGI1, CASPR2, AMPAR, GABA-A, GABA-B, DPPX, glycine receptor, VGCC, GAD65, MOG, ANCA, ENS2/ENC2 panels — all negative (serum and CSF) | MOG, GAD65, PCCA/ANNA, GABA-A, ENS2/ENC2 — all negative |
| **Index surgical/biopsy pathology** | Necrotizing encephalitis: perivascular + intraparenchymal lymphocytic infiltration, focal necrosis, gliosis, microgliosis; equivocal HSV IHC (2/3 stains negative); no intranuclear inclusions | CD8-predominant T-cell infiltrate, gray + white matter; reactive gliosis and microgliosis; no viral etiology, demyelination, PML, or MOGAD |
| **CD8 predominance confirmed** | Not formally established (repeat biopsy pending) | Yes |
| **Confirmed viral infection** | No (equivocal IHC; negative serial CSF PCR) | No |
| **Presumed mechanism** | Post-infectious / secondary cytotoxic autoimmune inflammatory injury | CD8+ cytotoxic T-cell–mediated cortical injury |
| **Diagnostic classification** | Chronic inflammatory encephalitis, etiology uncertain; RE spectrum possible | Probable late-onset Rasmussen's encephalitis (Bien criteria; atypical) |
| **ASM response** | Drug-resistant; focal aware seizures persisting on therapy | Drug-resistant (lamotrigine, levetiracetam, clobazam) |
| **Immunotherapy** | Empiric immunotherapy planned pending repeat biopsy | IV methylprednisolone + IVIG (peri-biopsy; unclear benefit); rituximab initiated (2 doses; well tolerated) |
| **Antiviral treatment** | Empiric valacyclovir (post-surgical; ongoing ID follow-up) | Not applicable |
| **Surgical intervention** | Right temporal craniotomy (December 2022); subsequent disease progression | None (lesion extent; absent hemiparesis); neuromodulation under consideration |
| **Follow-up status** | Progressive imaging; stable neurologic exam; repeat biopsy planned | Clinically stable on rituximab (January 2026); seizure burden monitoring ongoing |

*ASM, antiseizure medication; BBB, blood-brain barrier; BG, basal ganglia; FBTC, focal bilateral tonic-clonic; FIC, focal impaired consciousness; HSV, herpes simplex virus; IC, internal capsule; ID, infectious disease; IED, interictal epileptiform discharge; IHC, immunohistochemistry; LPD, lateralized periodic discharge; LRDA, lateralized rhythmic delta activity; OB, oligoclonal bands; PML, progressive multifocal leukoencephalopathy; RE, Rasmussen's encephalitis.*
