# Supplementary material for: Adult‐onset CD8‐predominant hemispheric inflammatory epilepsy: Expanding the spectrum of post‐infectious and Rasmussen‐like encephalitis
Source: Epileptic Disord. 2026 Jul 4;28(5):1679–82. doi: 10.1002/epd2.70281 (PMC13695466; doi:10.1002/epd2.70281)
Supplement: Supplementary file 3 [file EPD2-28-1679-s002.docx]

**TEST YOURSELF**

**Answers**

Q1	**Correct answer: B** Rationale: RE's hallmark is cytotoxic (cell-mediated) rather than humoral injury. Because no pathogenic antibody drives the process, even broad autoimmune panels return negative—making tissue biopsy essential for diagnosis.

Q2	**Correct answer: C** Rationale: Prospective data for tacrolimus and IVIG demonstrate slowing of progression but not reversal of drug resistance. Rituximab is an emerging option with limited follow-up data. Hemispherectomy evidence is predominantly pediatric.

Q3	**Correct answer: B** Rationale: Post-HSV anti-NMDAR encephalitis is antibody-mediated (humoral), emerging weeks after viral clearance and detectable serologically. In contrast, the cytotoxic RE-spectrum cases described here are seronegative with CD8^+^ T-cell predominance on tissue, a fundamentally different immunopathologic mechanism requiring biopsy for characterization.
